# Blood-based DNA methylation captures variance in adult height

**DOI:** 10.1186/s13059-025-03918-7

**Published:** 2026-01-20

**Authors:** Alesha A. Hatton, Robert F. Hillary, Daniel L. McCartney, Sarah E. Harris, Simon R. Cox, Kathryn L. Evans, Rosie M. Walker, Matthew Suderman, Paul Yousefi, Allan F. McRae, Riccardo E. Marioni

**Affiliations:** 1 Institute for Molecular Bioscience, The University of Queensland, Brisbane, QLD 4072 Australia; 2 Centre for Genomic and Experimental Medicine, Institute of Genetics and Cancer, University of Edinburgh, Edinburgh, EH4 2XU UK; 3 Lothian Birth Cohorts, Department of Psychology, University of Edinburgh, Edinburgh, EH8 9JZ UK; 4 School of Psychology, University of Exeter, Exeter, EX4 4QG UK; 5 Medical Research Council Integrative Epidemiology Unit at the University of Bristol, University of Bristol, Bristol, UK; 6 NIHR Bristol Biomedical Research Centre, University Hospitals Bristol and Weston NHS Foundation Trust and University of Bristol, Bristol, UK; 7 Population Health Science, Bristol Medical School, University of Bristol, Bristol, UK

**Keywords:** DNA methylation, Height, Methylation profile score

## Abstract

**Background:**

While height is a highly heritable trait with strong polygenic prediction, previous studies have postulated that minimal variation of its individual differences can be captured by DNA methylation (DNAm). We investigated the role of blood-based genome-wide DNAm in capturing the variance in adult height in a large population-based cohort of 7,654 unrelated individuals from Generation Scotland using DNAm profiled on the Illumina EPIC array. The posterior DNAm probe effects were used to construct a DNAm profile score (Methylation Profile Score—MPS) which was evaluated in three independent cohorts.

**Results:**

Genome-wide DNAm captures 25.0% (95% credible interval (CrI) 17.2–31.9) of the phenotypic variation in height when applying Bayesian penalised regression using BayesR + conditional on genetic effects. The total variation captured jointly by DNAm and genetic effects (80.3%, 95% CrI 70.1–87.2) is larger than the marginal estimate based on genetic effects only (56.3%, 95% CrI 45.8–66.8). Out-of-sample prediction shows that the MPS is weakly correlated with measured height (Pearson correlation ranging from 0.14–0.26), as well as being associated with several health and lifestyle factors in the LBC1936 that are established correlates of height.

**Conclusion:**

With the advent of larger sample sizes in epigenomics anticipated to improve the power to detect associations between DNAm and complex traits, we urge caution when making assumptions around “null traits” based solely on methylome-wide association study results and encourage the use of whole-genome methods to assess the proportion of variation in a trait that may be captured by DNAm.

**Supplementary Information:**

The online version contains supplementary material available at 10.1186/s13059-025-03918-7.

## Background

Height is one of the most heritable human quantitative traits [1], with the additive genetic contribution to adult height consistently estimated to be approximately 80% [2, 3]. The most recent genome-wide association study (GWAS) of 5.4 million individuals of diverse ancestries identified 12,111 independently associated SNPs, with 40% of the variation in height explained in an out-of-sample prediction in individuals of European (EUR) ancestry [4]. However, the 20% non-heritable component of height cannot be captured by these large genetic studies. Environmental factors have also been linked to variation in height [5], including nutrition [6], socio-economic status [7] and prenatal maternal weight [8]. Longitudinal studies have demonstrated that such factors have the greatest influence in early childhood. In contrast the genetic contribution increases with age, being greatest in adolescence [9].

DNA methylation (DNAm) is an epigenetic modification that is under both genetic and environmental influence and has been linked to exposure to external and lifestyle stressors such as smoking [10], body mass index (BMI) [11], nutrition [12] and prenatal risk factors [13]. Variation in DNAm patterns at height-associated genes (informed from GWAS loci) have been implicated in the mediation of environmental influences on height [14]. It is therefore possible that DNAm offers additional insights over genetics into the biological mechanisms underlying height. More recently, a methylome-wide association study (MWAS) of childhood height (*n* = 1,927) identified robust associations in three CpGs in the suppressor of cytokine signalling 3 (*SOCS3*) gene which were independent of genetic effects [15]. However, height has been previously considered to be a “null trait” in the context of DNAm associations. For example, Shah et al. found a methylation-profile score (MPS) accounted for almost no variation in height [16]. Further, Zhang et al. found that when jointly fitting DNAm probes and common genetic variants, DNAm captured none of the variance for height [17], with these results suggesting the prediction accuracy for height would not be improved by incorporating DNAm data.

Here, we investigated the role of blood-based genome-wide DNAm in capturing variation in height in a large, population-based cohort, Generation Scotland (GS, *n* = 7,654) using DNAm profiled on the Illumina EPIC array. We utilise Bayesian and restricted maximum likelihood approaches to estimate the proportion of variation in height captured by DNAm, both with and without the presence of common genetic effects. We construct a MPS for height and validate this in three independent cohorts. Lastly, we perform a phenotype-wide association study (PheWAS) between the MPS and health and lifestyle related outcomes in the LBC1936 to identify factors that may explain the association between DNAm and height.

## Results

### Study cohort

Blood-based DNAm and height were assessed in 7,654 unrelated individuals in the GS cohort as the discovery cohort, with out-of-sample prediction assessed in three independent cohorts (LBC1936 *n* = 861, LBC1921 *n* = 435, ALSPAC *n* = 5,628; Table 1). The GS cohort comprised of 56.3% females with a mean age of 51.6 years for all participants (SD 13.2, range 18–93 years). The mean height of participants was 168.0 cm (SD 9.5), with males (176.0 cm, SD 6.9) being taller than females (162.0 cm, SD 6.5). Plots of height and height by sex are shown in Additional file 1: Fig. S1. The three replication cohorts spanned the life course, with ALSPAC participants ranging from childhood to adulthood and the LBCs an older adult cohort (Table 1).

**Table 1:** Cohort characteristics for Generation Scotland (GS), Lothian Birth Cohorts (LBC1936 and LBC1921) and the Avon Longitudinal Study of Parents and Children (ALSPAC). ALSPAC participants included the offspring generation with collection at 7, 9, 15 or 17 and 24 years of age as well as the parental generation

Sample	N	Age (years), mean (SD)	Sex, N female (% female)	Height (cm), mean (SD)
Generation Scotland	7,654	51.6 (13.2)	4,311 (56.3%)	168.0 (9.5)
LBC1936	861	69.6 (0.8)	425 (49.4%)	166.4 (8.8)
LBC1921	435	79.1 (0.6)	263 (60.5%)	162.8 (9.2)
ALSPAC				
Age 7	914	7.5 (0.2)	460 (50.3%)	126.0 (5.2)
Age 9	343	9.8 (0.3)	173 (50.4%)	139.9 (6.2)
Age 15–17	2408	17.5 (0.8)	1255 (50.9%)	171.8 (9.6)
Age 24	752	24.4 (0.7)	367 (48.8%)	173.6 (9.1)
Parental generation (Mothers and Fathers)	1211	50.0 (5.4)	746 (61.6%)	169.8 (9.0)

### The proportion of variance in height captured by genome-wide DNAm

**Fig. 1:**
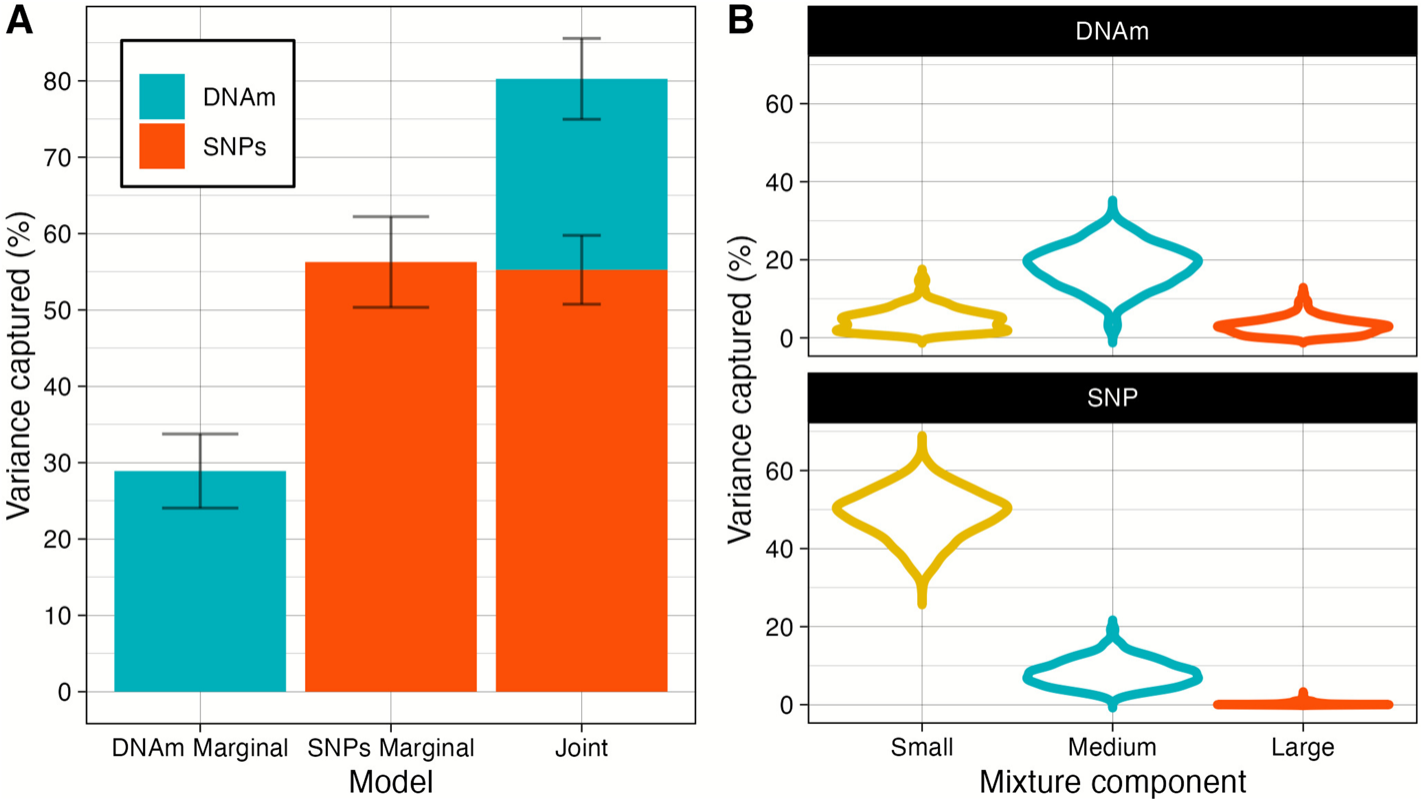
Variance component analysis of adult height in Generation Scotland. **A** The proportion of phenotypic variance in age-and-sex adjusted height captured by genome-wide DNAm (blue) marginally, SNPs (red) marginally and DNAm and SNPs jointly. Marginal models include genome-wide DNAm or SNPs only. Joint models fit both genome-wide DNAm and SNPs simultaneously i.e. when conditioned on one another. Variance estimates are presented for BayesR + approach. Error bars represent SE of the estimate. **B** Violin plot of the distribution of phenotypic variance attributable to components with small, medium and large effects for each DNAm and SNP (that capture 0.01%, 0.1% and 1% of the phenotypic variance, respectively) from the joint variance component analysis presented in part A

We implemented a Bayesian penalised regression method, BayesR +, that partitions trait variance under a model of polygenicity by modelling DNAm (and SNP) effects to be from a mixture of normal distributions. We set one of these mixtures to be a discrete spike at zero to allow for sparsity in estimated effects. Variance component analysis indicated that 28.9% (95% credible interval (CrI) 20.4–36.5) of the phenotypic variance was captured marginally by DNAm compared with 56.3% (95% CrI 45.8–66.8) by SNPs (Fig. 1A and Additional file 2: Table S1). The total variance captured when modelling both DNAm and SNPs jointly was estimated as 80.3% (95% CrI 70.1–87.2), with 25.0% (95% CrI 17.2–31.9) of the phenotypic variance captured by DNAm and 55.3% (95% CrI 46.2–63.3) by SNPs.

We used the omics-based restricted maximum likelihood (OREML) approach for sensitivity analyses, which assumed an infinitesimal model where DNAm (and SNP) effect sizes come from a single normal distribution. We found variance estimates to be largely concordant between the two methods (Additional file 1: Fig. S2 and Additional file 2: Table S1). When modelled jointly, 29.2% (95% confidence interval (CI) 21.9–36.5) of the phenotypic variance was captured by DNAm and 53.4% (95% CI 45.4–61.4) by SNPs, with these components jointly capturing 82.6% (95% CI 73.0–92.2) of the phenotypic variance in height. We conducted additional sensitivity analysis by performing OREML with covariate adjustment for the first 20 DNAm PCs and the first 20 genetic PCs. We find that models with and without these adjustments yield comparable estimates (Additional file 2: Table S1). Additionally, we adjusted the OREML regression for deciles of the Scottish Index of Multiple Deprivation (SIMD) which is a measure of socioeconomic status (SES). This also yielded comparable estimates with 34.8% (95% CI 26.8–42.9) of the phenotypic variance was captured by DNAm marginally (Additional file 2: Table S1).

We controlled for further potential genetic effects in the variance component analysis by adjusting for a PGS of height constructed from the latest GIANT height GWAS [4]. Incorporating the GIANT PGS as a fixed effect in the BayesR + variance component analyses attenuated the marginal variance estimates for both DNAm and SNPs (from 28.9% (95% CrI 20.4–36.5) to 22.1% (95% CrI 13.7–31.7) for DNAm and 56.3% (95% CrI 45.8–66.8) to 26.0% (95% CrI 0.41–48.1) for SNPs; Additional file 1: Fig. S3 and Additional file 2: Table S1). We estimated DNAm captured 21.4% (95% CrI 14.0–30.7) of the variance in height when conditioning directly on genetic effects and controlling for background genetic effects using a PGS. Given GS accounts for 18,000 of the 4 million EUR individuals in the GIANT height GWAS [4], we performed sensitivity analysis using an earlier iteration of the GWAS [18] and found comparable results (estimate of variance captured jointly by DNAm of 18.6% (95% CrI 10.3–28.5); Additional file 2: Table S1). This suggests that only part of the variation in height captured by DNAm is attributable to common genetic effects.

We investigated whether the contribution of DNAm to height was consistent across the sexes by estimating the degree of shared covariance in height between males and females captured by DNAm. We observe similar estimates in the variance captured by DNAm in height for males and females (30.8% (95% CI 18.5-43.1) for males and 30.7% (95% CI 20.1-41.2) for females), with the DNAm correlation between sexes for height suggesting there is no difference in the variance captured between the sexes (r_DNAm_ = 0.99, SE = 0.02, *p* = 0.18).

### Epigenetic architecture

BayesR + was used to quantify the number of DNAm loci and SNPs that contribute to trait variance when modelled jointly. The mean contribution to DNAm variance of components with small, medium and large effects (to allow for markers that account for 0.01%, 0.1% and 1% of the variation in height) were 5.6%, 22.0% and 3.6%, attributable to 1767, 700 and 17 DNAm probes, respectively (Fig. 1B). In contrast, the mean contribution to genetic variance of components with small, medium and large effects were 58.6%, 9.9% and 0.3%, to 8505, 154 and 2 SNP, respectively. This suggests that most DNAm probe associations for height are relatively small, but on average larger than genetic effects.

### DNAm loci associated with height

The BayesR + analyses identified two height-associated DNAm loci (cg07386640, cg09612304) with a posterior inclusion probability (PIP) greater than 95%, with no other DNAm loci having a PIP greater than 80% (Additional file 2: Table S2). Both DNAm loci were unique to the EPIC array and lie in regions of long non-coding RNA. *cis*-mQTLs were identified for both DNAm loci within a 1 Mb window of the DNAm probe (rs7747636 associated with DNAm levels at cg09612304 and rs57556107 for cg07386640) [19]. We queried whether either of these mQTLs have been previously identified to be associated with height or lie within height associated loci [4]. The mQTL for cg09612304, lies within the height associated loci surrounding rs874302 (spanning 153255888–153325888), while the mQTL for cg07386640 is not associated with height or within height associated loci. Despite being associated with height independent of genetic effects, when assessed in GS, both DNAm loci are weakly correlated with the GIANT PGS (*r* = −0.08, *p* = 0.006 for cg09612304; *r* = 0.1 *p* = 0.001 for cg07386640).

We queried both DNAm loci in the EWAS catalogue [20] (accessed 22 July 2024) and found previously reported associations between DNAm levels at cg07386640 and incident COPD [21], cancer treatment: asparaginase enzymes and anti-metabolites [22], as well as nominal associations with prevalent ischemic heart disease (self-report), prevalent COPD (self-report), incident lung cancer [21], C-reactive protein (CRP) levels [23] and smoking [24]. DNAm levels at cg09612304 was previously reported to be nominally associated with CRP levels [23] and CCL14 protein levels [25] (Additional file 2: Table S2).

### Out-of-sample DNAm prediction

Weighted linear MPS for height were constructed and applied to three independent cohorts (LBC1936 *n* = 861, LBC1921 *n* = 435, ALSPAC *n* = 5,628). The weights for each DNAm probe were the mean posterior effect size estimates from the joint BayesR + analyses with a corresponding PGS constructed using SNP effects (referred to as the GS PGS). The MPS was converted to the same scale as height (cm) by mean centring and scaling by the variance. The MPS was weakly correlated with height in both LBC cohorts (Pearson *r* = 0.26, *p* = 1.8 × 10^–4^ in LBC1936; *r* = 0.18, *p* = 2.2 × 10^–14^ in LBC1921; Additional file 1: Fig. S4). Correlations of similar magnitude were observed for each of the time points in ALSPAC (*r* = 0.21, *p* = 1.9 × 10^–10^ at 7 years, *r* = 0.17, *p* = 1.6 × 10^–3^ at 9 years, *r* = 0.15, *p* = 2.2 × 10^–13^ at 15–17 years, *r* = 0.23, *p* < 3.2 × 10^–10^ at 24 years and *r* = 0.14, *p* = 9.4 × 10^–7^ in parents). We estimated the difference in height between the top and bottom decile of the MPS and found a 3.6 cm difference in LBC1936 (*p* = 1.4 × 10^–4^).

**Fig. 2:**
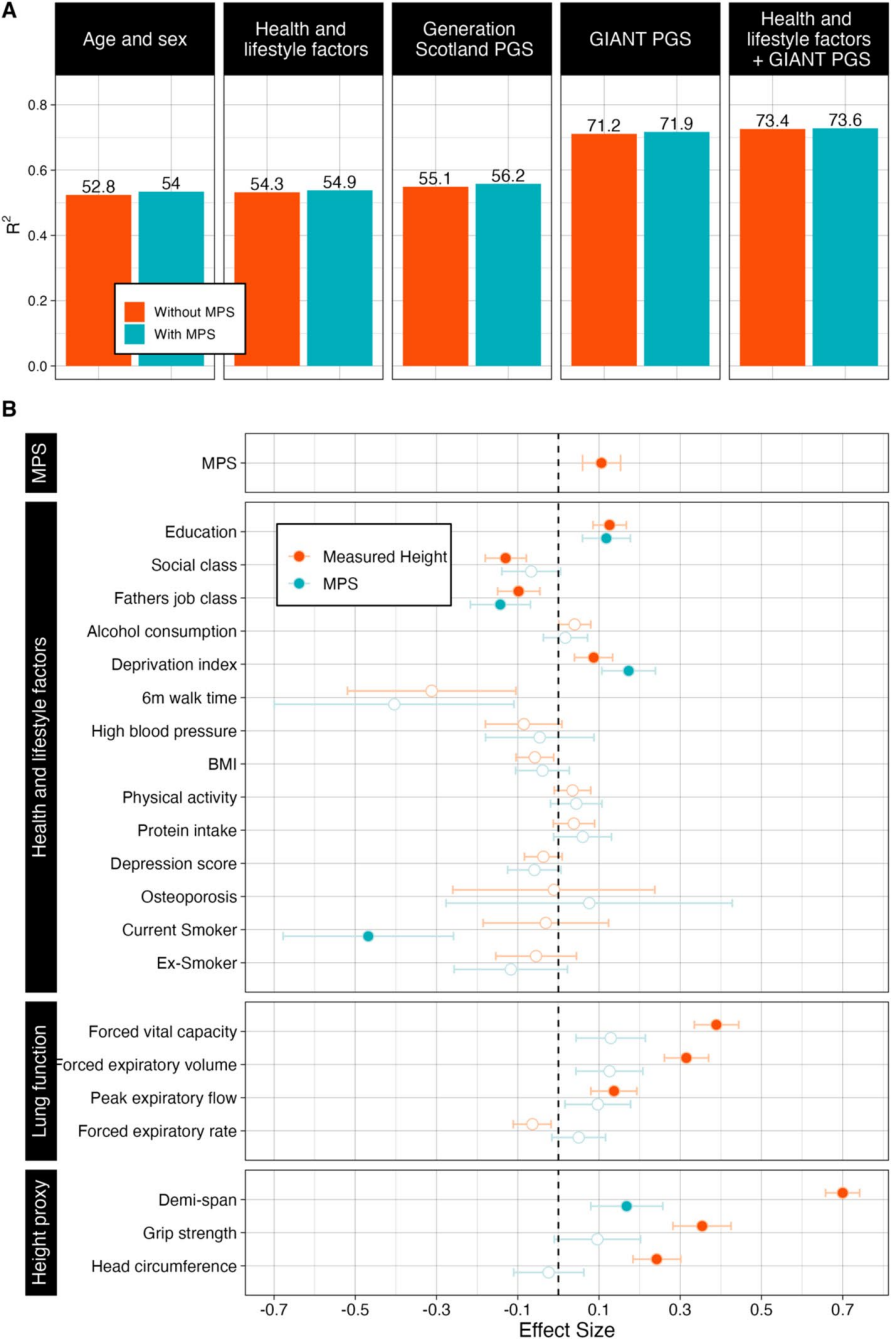
Prediction accuracy of the MPS and its association with health and lifestyle factors in LBC1936. **A** The prediction accuracy with the addition of the MPS for height measured in LBC1936 across five models. Performance of the different age- and sex-adjusted prediction models with (blue) and without (red) the inclusion of the MPS for height. Prediction accuracy was quantified using adjusted R^2^. The following models were considered (all models are adjusted for age and sex):(1) no additional covariates, (2) health and lifestyle factors, (3) GS PGS, (4) GIANT PGS (2022), (5) health and lifestyle factors and the GIANT PGS (2022). Health and lifestyle factors are listed in Additional file 2: Table S7. **B** The MPS for height and its association with health and lifestyle factors in LBC1936. Comparison of age- and sex-adjusted associations (Effect Size and *p*value) between the MPS and height with health and lifestyle factors. Coloured dots denote factors that were significant after Bonferroni correction for multiple testing. Error bars indicate 95% confidence intervals. All covariates were mean centred and scaled to unit variance.

We assessed the predictive ability of the MPS, obtaining an incremental R^2^ of 1.0% (*p*value of MPS = 9.2 × 10^–6^) in LBC1936 and 0.1% (*p* = 0.2) in LBC1921 (Fig. 2 and Additional file 2: Table S3). With the addition of the GS PGS, there was minimal change in the incremental R^2^, 0.9% (*p* = 4.0 × 10^–5^) in LBC1936 and 0.1% (*p* = 0.2) in LBC1921. When instead adjusting for the PGS based on GIANT, the prediction accuracy decreased to 0.5% (*p* = 5.5 × 10^–5^) in LBC1936, with a small increase to 0.3% (*p* = 0.03) in LBC1921. Given the decrease in MPS prediction upon addition of the PGS, we assessed the correlation between the MPS and the GIANT PGS and find no evidence of a correlation between the two (*r* = 0.06, *p* = 0.10 in the LBC1936 and *r* = −0.03, *p* = 0.60 in the LBC1921).

In ALSPAC, the prediction accuracy of the MPS was largest at the younger time points (incremental R^2^ of 3.6% and 2.4% at the 7 and 9 year follow up time points). The MPS explained a smaller percentage of the variation at later time points (0.1%, 1.0% and 0.2% at the 15-17 and 24 year time points and in parents, respectively; Additional file 2: Table S4).

Due to the effects of increasing age on height and the average age of the LBC cohorts (mean age 70 years in LBC1936 and 79 years in LBC1921), we also assessed the correlation of the MPS with demi-span (*r* = 0.24, *p* < 0.001 in LBC1936 and *r* = 0.15, *p* = 0.001 in LBC1921) and found similar results to that for height. In the LBC1936 we compared the associations between height and the MPS with each of head circumference, grip strength and four measures of lung function (forced vital capacity (FVC), forced expiratory volume (FEV), peak expiratory flow (PEF) and forced expiratory rate (FER)) which may also act as proxies for height. While these factors were associated with height, they were not associated with the MPS, despite displaying directionally consistent, but weaker associations (except for FER; Fig. 2 and Additional file 2: Table S5). We examined the association between DNAm predicted blood cell proportions (Bcell, CD4T, CD8T, Eos, Mono, Neu, NK) and the MPS in the LBC36 and LBC1921 and find no association between the MPS and cell type proportions (Additional file 2: Table S6).

### Phenome-wide association study (PheWAS) with MPS

We conducted a PheWAS of the MPS in the LBC1936 to identify associated covariates including 20 phenotypes, encompassing three subgroups: those broadly associated with health and lifestyle factors, lung function and proxies of measured height (Additional file 2: Table S7). We compared associations for each of the health and lifestyle factors between those observed for the height MPS and measured height. In age- and sex- adjusted linear regression analyses (after Bonferroni correction, *p* < 0.05/20 = 0.0025) the MPS was associated with years of education, father’s job class, deprivation index and smoking status (current smokers) (Fig. 2 and Additional file 2: Table S5). Of note, smoking status (current smokers) was associated with the MPS but not with measured height. When jointly adjusting for all health and lifestyle factors (see health and lifestyle factors in Additional file 2: Table S7), the MPS remained a small but significant predictor of height (*p* = 3.5 × 10^–3^; incremental R^2^ of 0.05%; Fig. 2, Additional file 2: Table S3 and Table S8). This decreased upon addition of the GIANT PGS (*p*value of MPS = 0.02; incremental R^2^ of 0.02%).

## Discussion

In the context of investigating phenotypic variation captured with blood-based DNAm, adult height has previously been considered a “null trait” [16, 17]. Utilising a large-scale population cohort, we demonstrated a substantial proportion of the variation in height can be captured with DNAm, both with and without the presence of common genetic effects. We demonstrate the robustness of this result by adjusting for a PGS of height and find minimal attenuation of the DNAm variance. Zhang et al. previously estimated no variation in height was captured by DNAm when jointly fitting DNAm probes and common genetic variants using the OREML approach [17]. We suggest the variance estimate presented in that study was likely the result of limited statistical power due to sample size (*n* = 1,342). This conclusion is supported by the lower DNAm variance estimate for BMI also reported in that study compared to more recent studies with larger sample sizes. For example, Zhang et al. estimated that DNAm captured 6.5% (SE 3.8%) [17] of the variance in BMI while several larger studies utilising GS obtained estimates of 59.5—76.7% (SE 2.0- 2.7%) [26–28].

We developed a MPS and show this is correlated with height in three independent cohorts, but with very low out-of-sample prediction accuracy. We found the largest percentage variance explained by the MPS was in the younger follow up time points in ALSPAC (3.6% at 7 years and 2.4% at 9 years). This temporal pattern suggests DNAm may be capturing elements of early growth associated with height that potentially reflect environmentally mediated influences on height that are strongest in early life. Similar age -dependent effects were reported by Issarapu et al., who found that DNAm at *SOCS3* were strongest in early life (between birth and 5 years), while genetic effects increase from birth to 21 years, consistent with the declining impact of environmentally responsive DNAm marks over the life course [15]. Our EWAS results did not replicate any of the reported CpG findings in *SOCS3*, however this has reported to only be strongly associated with height between birth and 5 years and our discovery dataset comprised of adults. These findings underscore the potential of DNAm as a biomarker of early environmental exposures that influence growth with future work integrating repeated DNAm measures to track stability or changes in epigenetic signals over development, potentially informing interventions for growth-related traits.

In an earlier study by Shah et al., a MPS of height explained 0.3% and 0.8% (*p*value = 0.02 and 0.01) of the variation in out-of-sample prediction in the LBCs (*n* = 1,366) and LifeLines DEEP (*n* = 752) cohorts, respectively which is in line with that reported here (when accounting for the smaller discovery sample) [16]. We recognise that low prediction accuracy in the LBCs may be due to dysregulation of blood-based DNAm as a result of aging, with a known decline in average DNAm levels with increasing age [29, 30]. Additionally, this may be due to difficulties in obtaining accurate height measurements in older individuals. This would also explain the differences in prediction accuracy observed between LBC1936 and LBC1921 (note a 3 cm difference in mean height between the LBC cohorts). However, the weak correlation between the MPS and height, as well as similarity in magnitude of correlation with demi-span, suggests the MPS is capturing relevant variation. Similarly, we investigated the accuracy of the MPS in predicting head circumference which both can act as a proxy for intracranial volume (ICV) and thus maximum healthy brain size, as well as providing an indication of body size in an ageing population which may have reduced vertical height. We found no association with the MPS, providing evidence against DNAm capturing confounding variation associated with cognitive functioning (as larger ICV has been shown to be moderately related to higher general cognitive functioning [31]). This suggests the MPS is capturing non-brain (and cognitive)-related variance in traits like education. While systematic differences in the DNAm patterns between GS, the LBCs and ALSPAC may be responsible for part of the poor MPS performance, previous studies on C-reactive protein levels [23] and alcohol consumption [32] observe stronger performance of DNAm based predictors when following a similar study design than we find for height. This indicates that the poor performance of the MPS for height is being driven by the epigenetic architecture of the associations with height that align more closely to the infinitesimal model.

In our analyses, the variance captured by DNAm and genetic effects was only minimally attenuated when both were modelled jointly, consistent with these effects being largely independent. However, the predictive utility of the MPS beyond the PGS was limited, with the out-of-sample prediction of the MPS attenuated when conditioned on the GIANT PGS. This suggests incomplete separation of the genetic and epigenetic signals. Given that the external GIANT PGS was derived from a much larger discovery sample, it may capture a broader genetic architecture that the in-sample SNPs used in our variance decomposition. Further, despite being associated with height independent of genetic effects, the two DNAm loci identified here have known mQTLs and therefore additionally capture genetic influence. It is therefore not unexpected that the MPS retains some genetic contribution, even when the weights are estimated conditional on genetic effects. These findings indicate that while the DNAm component predominantly captures environmentally mediated variance, it may still reflect genetic variation.

The low predictive ability of the MPS, despite DNAm capturing 25.0% of the phenotypic variation in height, suggests the MPS will improve when using training datasets with larger sample sizes (in individuals of similar age to GS). We note that early on in GWAS, with studies of small sample sizes, there too was limited predictive ability for many complex traits. With the increase in sample size for GWAS, now into the millions [4], prediction for most complex traits have improved. Given DNAm probe effect sizes for height were larger than genetic effects, such improvement may be attained with a relatively smaller increase in sample sizes compared to GWAS, perhaps requiring hundreds of thousands of individuals rather than millions. This conclusion is consistent with the identification of only two DNAm loci with PIP > 0.8, whereby limited accuracy for true but weak effects likely contribute to the low out-of-sample prediction. We expect that larger sample sizes are required for sufficient power to detect individual probe associations with height. Lastly, we demonstrate the MPS is associated with several health and lifestyle factors that are established correlates of height. Further identification of individual probe associations would also allow for the causal nature of such relationships to be explored (e.g. DNAm may be mediating the relationship between these exposures and height, or they may be associated through horizontal pleiotropy).

This study is strengthened by the use of two robust variance partitioning approaches. Both BayesR + and OREML have been shown to be robust to various potential sources of heterogeneity and confounding and provide unbiased point estimates [33]. We recognise that DNAm arrays capture a small portion of the methylome and may provide an incomplete or biased view of the contribution of DNAm to phenotypic variation, particularly given the contribution of rare SNPs to height [34]. While a relatively large proportion of the phenotypic variance was captured in whole blood, the blood methylome has been shown to display a distinctive profile compared with other somatic tissues [35]. Thus, replication in a more trait-relevant tissue such as skeletal bone or muscle may capture more biologically pertinent associations.

## Conclusion

These efforts demonstrate that substantial variation in height is captured by DNAm. As was demonstrated with GWAS, the advent of large sample sizes in epigenomics will lead to improved power to detect associations between DNAm and complex traits, even for traits that have been considered “null traits” in studies involving small sample sizes. Accordingly, we urge caution when making assumptions around “null traits” based solely on MWAS results and encourage the use of whole-genome methods (e.g. OREML and BayesR +) to assess the proportion of variation in a trait that may be captured by DNAm.

## Methods

### Study cohort

The GS cohort is a family-based genetic epidemiological cohort that consists of over 24,000 volunteers, as described elsewhere [36, 37]. Recruitment took place between 2006 and 2011, when individuals and their family members were invited to a baseline clinic visit that included health questionnaires and sample donation for genomic analyses. Height was measured at the clinic visit to the nearest half centimetre. For the present analyses, height was adjusted for age, age squared and sex using linear regression. The residuals from this model were entered as a dependent variable in the subsequent analysis. Genome-wide blood-based DNAm was assessed using the EPIC array with DNAm QC presented in the supplemental methods (Additional file 1). Information on genotyping for is presented in the supplemental methods. After filtering, this study uses phenotypic, DNAm and genetic data from unrelated samples (*n* = 7,654, based on GRM < 0.05). Before analysis, DNAm at each CpG site was adjusted for age, sex, batch, slide, cell type proportions [38] and epigenetic predicted smoking status [39].

### Variance component analysis and MWAS

Bayesian penalised regression using BayesR + [28] was employed to simultaneously estimate the variance explained in height by DNAm and to identify individual probes that were associated with height. We further used linear mixed model regression performed using OREML applied in the OSCA software [17] as a sensitivity analysis to estimate the proportion of phenotypic variance in height captured by genome-wide DNAm. We corrected for further potential genetic influence by adjusting for a PGS of height constructed from the latest GWAS of height based on sample of European ancestry only (GIANT PGS) using SBayesC [4, 40]. Lastly, we employed a bivariate variance decomposition approach to assess the degree of shared contribution of DNAm to height between sexes [26]. Full details of statistical methods are provided in the supplemental methods.

### DNAm prediction

A weighted linear MPS of height was evaluated in three independent cohorts. The weights for each DNAm probe were the mean joint posterior effect size estimates from the BayesR + analyses in GS. These weights were applied to DNAm samples from three cohorts for individuals with concurrent DNAm, SNPs and height measurements: the Lothian Birth Cohort of 1921 (LBC1921) and 1936 (LBC1936), and the Avon Longitudinal Study of Parents and Children (ALSPAC) (see supplemental methods for cohort descriptions).

### PheWAS with MPS

We conducted a PheWAS of the MPS in the LBC1936 to identify associated covariates including 20 phenotypes, encompassing three subgroups: those broadly associated with health and lifestyle factors, lung function and proxies of measured height (Additional file 2: Table S7). We regressed the MPS (unit in cm) on each of the phenotypes, including adjustment for age and sex and compared this with a similar regression using measured height as the dependent variable. In addition, measured height was regressed on the MPS.

## Supplementary Information

Additional file 1: Supplementary methods include cohort descriptions for Generation Scotland, The Lothian Birth Cohorts of 1921 and 1936, and Avon Longitudinal Study of Parents and Children, and description of methods for Bayesian penalised regression, Mixed model regression and PGS for height. Fig. S1. Density plots of height in Generation Scotland (GS), the LBC1921 and LBC1936. Fig. S2. The proportion of variance captured in height in Generation Scotland by genome-wide. Fig. S3. Variance captured in height in Generation Scotland by DNAm and SNPs after adjusting for a PGS of height by method. Fig. S4. Scatter Plot of height (measured) and MPS in the LBC1936 and LBC1921 [45–72].

Additional file 2: Table S1. Variance captured in height in Generation Scotland by genome-wide DNAm and SNPs by variance decomposition method. Table S2. Identification of DNAm probes associated with height in Generation Scotland. Table S3. Prediction accuracy of MPS for height in the LBC1936 and LBC1921. Table S4. Prediction accuracy of MPS for height in ALSPAC for each time point. Table S5. Marginal association of health and lifestyle, lung function and height proxy traits with height and MPS in the LBC1936. Table S6. Association between the MPS and DNAm predicted cell type proportions in the LBC1936 and LBC192. Table S7. Demographic health and lifestyle phenotypes in the LBC1936. Table S8. Joint association of health and lifestyle with height, with and without adjustment for MPS in the LBC1936.

## Data Availability

Researchers wishing to access the DNAm resource and wider Generation Scotland study data can do so by submitting an access application form to access@generationscotland.org (contact person Dr D. McCartney). Access applications are subject to review through GS access processes, which ensure that all research using the resource aims to benefit the health and wellbeing of patients and the public. Approved projects are subject to a Data & Materials Transfer Agreement (DMTA) or commercial contract. Full information on the access procedure including application forms and DMTA templates is available from the Generation Scotland website [41]. Data dictionaries describing the full GS resource are available online [42]. Instructions for accessing Lothian Birth Cohort data, alongside a Data Request Form template, Data Summary Tables and Data Dictionaries is available from the Lothian Birth Cohort website [43]. Completed Data Request Form applications are subject to review and a Data and/or Material Transfer Agreement. The ALSPAC study website contains details of all the data that is available through a fully searchable data dictionary and variable search tool [44]. We used publicly available software tools for all analyses. The following summary level data was used in this study: GIANT consortium data files [4]; DNA methylation QTLs [19].
