# Supplementary material for: Blood-based DNA methylation captures variance in adult height
Source: Genome Biol. 2026 Jan 20;27:37. doi: 10.1186/s13059-025-03918-7 (PMC12905977; doi:10.1186/s13059-025-03918-7)
Supplement: Supplementary file 1 — Additional file 1: Supplementary methods include cohort descriptions for Generation Scotland, The Lothian Birth Cohorts of 1921 and 1936, and Avon Longitudinal Study of Parents and Children, and description of methods for Bayesian penalised regression, Mixed model regression and PGS for height. Fig. S1. Density plots of height in Generation Scotland (GS), the LBC1921 and LBC1936. Fig. S2. The proportion of variance captured in height in Generation Scotland by genome-wide. Fig. S3. Variance captured in height in Generation Scotland by DNAm and SNPs after adjusting for a PGS of height by method. Fig. S4. Scatter Plot of height (measured) and MPS in the LBC1936 and LBC1921 [45–72]. [file 13059_2025_3918_MOESM1_ESM.docx]

Blood-based DNA methylation captures variance in adult height

Alesha A Hatton^1, †,^ *, Robert F Hillary^2, †^, Daniel L McCartney^2^, Sarah E Harris^3^, Simon R Cox^3^, Kathryn L Evans^2^, Rosie M Walker^2,4^, Matthew Suderman^5,6,7^, Paul Yousefi^5,6,7^, Allan F McRae^1,†^, Riccardo E Marioni^2,†^

^†^equal contributions (joint first and last authors)

Affiliations

1. Institute for Molecular Bioscience, The University of Queensland, Brisbane, QLD 4072, Australia
2. Centre for Genomic and Experimental Medicine, Institute of Genetics and Cancer, University of Edinburgh, Edinburgh, EH4 2XU, UK
3. Lothian Birth Cohorts, Department of Psychology, University of Edinburgh, Edinburgh, EH8 9JZ, UK
4. School of Psychology, University of Exeter, Exeter EX4 4QG, UK
5. Medical Research Council Integrative Epidemiology Unit at the University of Bristol, University of Bristol, Bristol, UK
6. NIHR Bristol Biomedical Research Centre, University Hospitals Bristol and Weston NHS Foundation Trust and University of Bristol, Bristol, UK
7. Population Health Science, Bristol Medical School, University of Bristol, Bristol, UK

*corresponding author

Email: [a.hatton@uq.edu.au](mailto:a.hatton@uq.edu.au) ORCID: 0000-0003-3564-4052

Postal address: 306 Carmody Rd, St Lucia QLD 4067

Table of Contents

[Supplemental methods 2](#_Toc215491664)

[Generation Scotland 2](#_Toc215491665)

[The Lothian Birth Cohorts of 1921 and 1936 3](#_Toc215491666)

[Avon Longitudinal Study of Parents and Children 4](#_Toc215491667)

[Bayesian penalised regression 5](#_Toc215491668)

[Mixed model regression 6](#_Toc215491669)

[PGS for height 7](#_Toc215491670)

[DNAm prediction 7](#_Toc215491671)

[Supplemental Figures 8](#_Toc215491672)

[References 12](#_Toc215491673)

## Supplementary methods

### Generation Scotland

#### Phenotype preparation

Height was measured at the clinic visit by asking volunteers to remove their shoes and to stand with their heels together, feet angled at 60 degrees and as tall as possible with their shoulders and back against the freestanding measurement device. The volunteers’ heads were held in the Frankfort horizontal plane, where the inferior border of the bony orbit is in line with the groove at the top of the tragus of the ear. During quiet breathing, height was measured to the nearest half centimetre with the horizontal arm of the measurement unit kept at a right angle to the scale.

The PheWAS assessed 20 phenotypes, encompassing three subgroups: those broadly associated with health and lifestyle factors, lung function and proxies of measured height (Additional file 2: Table S7). For BMI, six-meter walk and alcohol consumption, a log transformation was used due to a skew in the distributions, with a constant of one was added to the latter prior to log-transforming owing to zeros in the data. Prior to running the PheWAS, continuous variables were transformed by mean centering and unit variance scaling.

#### Genotype imputation and quality control

GS genome wide genotypic details have been described previously (45). Participants were genotyped with Illumina HumanOmniExpressExome8v1-2_A or HumanOmniExpressExome-8v1_A arrays. SNPs were excluded for missing genotype call rate (>2%), marked departure from HWE (p < 1 × 10^−6^) and low MAF (<1%). Duplicate samples were removed alongside individuals with gender mismatch and missing genotype call rate (>2%). PCs were calculated in the GCTA software (46) and projected against 1,092 individuals of the 1000 Genomes reference population (phase 1) (47). Ancestry outliers were defined as observations more than six standard deviations away from the mean of the EUR population cluster for the first two PCs and were subsequently removed (48). Genotype data were imputed against the TOPMed imputation reference panel (49) through the NHLBI BioData Catalyst at the TOPMed Imputation Server (<https://imputation.biodatacatalyst.nhlbi.nih.gov/>). Unrelated individuals were identified based on genomic relatedness (GRM threshold < 0.05 using the GCTA software). All subsequent analyses were conducted using HapMap3 (HM3) SNPs (n=1,189,629). Genomic positions are reported on build GRCh37/hg19.

#### DNAm quality control and normalisation

Genome-wide blood-based DNAm was assessed using the EPIC array as reported previously (50). This was conducted in three analysis sets. Quality control measures, described fully in Walker et al. (50), were implemented to exclude poor-performing probes and samples, as well as samples for which there was a mismatch between methylation-predicted sex and self-reported sex. Probes were also excluded if they targeted non-autosomal CpG sites, CpGs that were predicted to cross-hybridise or those with polymorphisms at the target site which can alter probe binding (51, 52). Probes with almost invariable beta values across individuals (standard deviation < 0.02) were excluded from the analysis. Participants with unreliable self-report disease data (i.e., replied yes to all possible options), saliva instead of blood DNA samples and potential XXY genotype were excluded. All 3 sets were normalised together with the final discovery dataset comprising M values at 752,439 loci for 7,654 participants. Before analysis, DNAm at each CpG site was adjusted in the OSCA software for age, sex, batch (to account for batching of samples for methylation array processing), slide (to correct for samples included on the same slide), cell type proportions (estimated using the algorithm proposed by Houseman et al. (53)) and epigenetic predicted smoking score (EpiSmokEr (54)).

### The Lothian Birth Cohorts of 1921 and 1936

#### Cohort description

The Lothian Birth Cohort of 1936 (LBC1936) and 1921 (LBC1921) are longitudinal studies of aging in the Edinburgh area of Scotland, focusing on lifetime cognitive change (55, 56), containing biomarker, genetic, DNAm and phenotypic data (<https://www.ed.ac.uk/lothian-birth-cohorts>). The LBC1936 and 1921 cohorts are follow-up studies of the Scottish Mental Health Surveys of 1947 and 1932, with subjects at an average age of 70 years (SD 0.8) and 79 years (SD 0.6), respectively. An overview of the data collected can be found in the cohorts’ profile article (55, 57) and has been described extensively elsewhere (58, 59). We included in the analysis only the first wave of the LBC data.

#### DNAm quality control and normalisation

We considered blood-based DNAm data from LBC1921 and LBC1936 cohorts. For both cohorts all individuals had DNAm assayed on Illumina HumanMethylation450 BeadChip. Normalisation and quality control of the DNAm data were performed independently for each cohort using the R package meffil (60). Standard QC threshold parameters were used to remove individuals with outlying methylated/un-methylated ratios, high missing calls, discordance between reported and estimated sex, and probes with excess missingness. Predicted cell counts (Bcell, CD4T, CD8T, Mono, Neu and NK) were calculated using the Houseman algorithm implemented in meffil (53). Probes on the sex chromosomes or those annotated as binding to multiple sites across the genome were excluded (61). After cleaning, 404,503 DNAm probes which were common across all cohorts remained for association analysis. The current study used a subset from this dataset comprising 374,824 CpGs (overlapping with those included in the Generation Scotland analyses) for 861 LBC1936 (435 LBC1921) individuals.

#### Genotype imputation and quality control

Genotyping was performed for both cohorts using the Illumina 610-Quad Beadchip array, with SNP genotype QC performed following standard protocols (62). Genotype data were restricted to autosomes and SNPs were excluded for missing genotype call rate (>5%), marked departure from Hardy–Weinberg equilibrium (HWE; p < 10−6), low minor allele frequency (AF<1%) and deviance in minor AF from 1000Genomes reference panel European samples (47). QC exclusionary measures for subjects were: missing genotype call rate (>5%); suspected sample error or contamination indicated by high heterozygosity or indeterminate genetic sex; ancestral outliers and genomic relatedness (> 5%). SNPs were phased using EAGLE2 (v2.0.5)+PBWT (63) and imputed against 1000Genomes Phase 3 reference panel (47) using Sanger Imputation Service. Imputed SNPs were then filtered based on info score (< 0.8), HWE (p < 10−6) and minor AF (<1%). There were 8,297,026 SNPs remaining post imputation and QC.

### Avon Longitudinal Study of Parents and Children

#### Cohort description

The ALSPAC cohort is a transgenerational prospective study of the genetic and environmental characteristics that influence health and development in parents and their children, that has been described in detail previously (64-67). Briefly, participants comprise of a cohort of offspring born to pregnant women resident in Avon, UK with expected dates of delivery 1st April 1991 to 31st December 1992. Participating offspring, mothers, and fathers were followed through a series of ongoing data collection waves that formed a detailed biobank including DNA and a wide range of biospecimen assays. Study data were collected and managed using REDCap electronic data capture tools hosted at the University of Bristol (68). REDCap (Research Electronic Data Capture) is a secure, web-based software platform designed to support data capture for research studies. DNA methylation was measured in the peripheral blood of the offspring at ages 7, 9 and 15–17 and of their mothers during pregnancy and approximately 18 years later (69). Details of how to access these data are provided in the cohort profile (69). The analytical sample in our study included 1,211 parents who had genetic, DNAm and height measured 18 years after recruitment (746 mothers and 465 fathers) and four longitudinal measurements of DNAm and height as well as genetic data were available for the children at the following ages: 7, 9, 15 or 17 and 24 years. The number of samples available at each time-point was 914, 343, 2408 and 752. DNAm was profiled using the 450k array for measurements in the parents as well as at time points 7 and 9 and for 894 of the individuals at the 15 or 17 year time point with 384,672 DNAm sites available for testing. For the remaining 1514 individuals with DNAm profiled at the 15 or 17 year time point and those with measurements at the 24 year time point, these were profiled using the EPIC array with 749,470 DNAm sites available for testing.

#### DNAm quality control and normalisation

Methods for methylation measurements in ALSPAC have been described previously (69). Briefly, peripheral blood (whole blood or buffy coats) was collected according to standard procedures, spun, and frozen at -80˚C. DNA methylation assays and data pre-processing was performed at the University of Bristol as part of the ARIES project. DNA was extracted using standard protocol and was bisulfite-converted using the Zymo EZ DNA MethylationTM kit (Zymo, Irvine, CA). DNA methylation was then measured using the Infinium HM450 or EPIC BeadChip assay (Illumina Inc, San Diego, CA), according to the standard protocol. Arrays were scanned using an Illumina iScan. An initial review of data quality was assessed using GenomeStudio (version 2011.1). A semi-random approach (sampling criteria were in place to ensure that all time points were represented on each array) was used to distribute ARIES samples across slides to minimize the possibility of potential confounding by batch. Data were pre-processed and normalised using the meffil R package (60).

#### Genotype imputation and quality control

ALSPAC participants had been previously genotyped as part of the larger ALSPAC study, with QC, cleaning and imputation performed at the cohort level before extraction of the subset analyzed in this study. ALSPAC offspring were genotyped at the Wellcome Trust Sanger Institute, Cambridge, UK and the Laboratory Corporation of America, Burlington, NC, USA using the Illumina HumanHap550 quad chip array. ALSPAC mothers were genotyped at Centre National de Génotypage, Paris, France, using the Illumina Human 660W-quad array and genotypes were called with Illumina GenomeStudio. ALSPAC fathers were genotyped at the ALSPAC Laboratory, Bristol, UK, using the Illumina HumanCoreExome array. SNPs with call rate <95%, lack of HWE (P <5e-7) or MAF <1% were excluded. Individuals with gender mismatches, minimal or excessive heterozygosity, missingness >3%, insufficient sample replication (IBD<0.8) or potential ID mismatches were excluded. Population stratification was assessed by MDS and compared with Hapmap phase 2 reference populations; all individuals with non-European ancestry were removed. For ALSPAC, array genotypes were harmonized, phased using SHAPEIT v2 (70) and subsequently imputed via the Michigan imputation server (71) to the Haplotype Reference Consortium (HRC) reference panel (72) (for mothers and children) or to the 1000 Genomes phase 1 version 3 reference panel (3) (for fathers).

Bayesian penalised regression

Bayesian penalised regression with Gaussian mixture-based variance partitioning was performed using the BayesR+ software (29). This was used to simultaneously estimate the variance explained in height by DNAm and to identify individual probes that were associated with height. Gaussian priors were specified to accommodate small, medium, and large effect sizes (mixture variances of 0.0001, 0.001, and 0.01) to allow for markers that account for 0.01%, 0.1% and 1% of the variation in height, respectively. A discrete spike at zero allowed for the incorporation of markers with non-identifiable effects. The joint and conditional probe effects were estimated after scaling both the outcome (residuals from a linear regression model of height on age, age squared and sex) and predictors (DNAm corrected for covariates described above using linear regression models for each of the 752,439 CpG sites) to mean zero and unit variance. A combined regression was performed with DNAm and genotype data, with each SNP scaled to mean zero and unit variance, in order to jointly estimate the variance explained in height by DNAm and SNPs. The same three prior variances were set for each DNAm and SNPs. The Gibbs sampling procedure is described in the supplementary methods.

Gibbs sampling was performed to sample over the posterior distribution conditional on the input data to obtain variance components estimates as well as individual DNAm and SNP associations with height from the BayesR+ model (29). Across 10,000 iterations, every 5th sample (to reduce autocorrelation) was retained. This was repeated over four chains after specifying different random seeds. The last 250 iterations from each chain were combined for downstream analyses. The variance component estimates were taken as the mean across the 1000 iterations with the 2.5% and 97.5% percentiles forming the 95% credible interval and DNAm probes with a posterior inclusion probability (PIP) of 95% considered significant.

Mixed model regression

We employed linear mixed model regression as a sensitivity analysis to estimate the proportion of phenotypic variance in height captured by genome-wide DNAm. This was performed using OREML applied in the OSCA software (30). This method estimates the variance captured by DNAm through construction of a methylation relationship matrix (MRM) based on all DNAm probes which is used to model the covariance between individuals in a univariate linear mixed model via REML. The genetic relationship matrix (GRM) was used to estimate the proportion of phenotypic variance explained by all SNPs (SNP-based heritability). These quantities were also jointly estimated in the OSCA software using --multi-orm which allows for multiple random effects (omics matrices). Further sensitivity analyses were performed with covariate adjustments for the first 20 PCs of the DNAm levels and the first 20 PCs of the genetic data. We used a bivariate variance decomposition approach to assess the degree of shared contribution of DNAm to height between the sexes (31). We estimate the DNAm correlation between the sexes for height, implemented using the bivariate OREML framework in OSCA. This approach is akin to estimating genetic correlations between the sexes (32) and estimates the shared contribution of DNAm to height based on the MRM between sexes. A likelihood ratio test was performed to test the hypotheses of fixing the correlation at one.

PGS for height

We corrected for further potential genetic influence by adjusting for a PGS of height as a covariate in both the BayesR+ and OREML analyses. SBayesC weights (33) from GIANT (34) (EUR samples only) were downloaded from <https://cnsgenomics.com/data/giant_2022/>, with the PGS constructed using plink (35). Given GS was included in the GIANT discovery GWAS (accounting for ~18,000 of the 4 million samples of EUR ancestry) this may bias prediction, albeit likely minimally. We therefore performed sensitivity analysis by constructing a PGS from an earlier GWAS from GIANT (36). The PGS was constructed by applying the SBayesC method (implemented in the GCTB software (37)) to the GWAS summary statistics with a shrunk, sparse LD matrix for HM3 SNPs calculated from UK Biobank samples of EUR ancestry (33).

DNAm prediction

A weighted linear MPS of height was constructed in three independent cohorts. Scores were either comprised of weights from all EPIC probes or those probes that overlapped between the EPIC array and the 450K array, dependent on the profiling of the samples. The weights for each DNAm probe were the mean joint posterior effect size estimates from the BayesR+ analyses in GS, with a corresponding PGS constructed using joint SNP effects (GS PGS). These weights were applied to DNAm samples from three cohorts for individuals with concurrent DNAm, SNPs and height measurements: the Lothian Birth Cohort of 1921 (LBC1921) and 1936 (LBC1936), and the Avon Longitudinal Study of Parents and Children (ALSPAC). The MPS was converted to the same scale as height (cm) by mean centring and scaling by the variance. Linear regression was used to assess the association between the MPS and measured height (adjusted for age and sex) in each cohort. We quantified the prediction accuracy of the MPS as the difference between the variance explained (adjusted R^2^) by the full linear model of MPS and covariates minus that explained by a reduced linear regression model not including the MPS (the difference being termed the incremental R^2^). The following models were considered:

1. Height ~ age + sex + MPS
2. Height ~ age + sex + GS PGS + MPS
3. Height ~ age + sex + GIANT PGS + MPS
4. Height ~ age + sex + health and lifestyle factors + MPS
5. Height ~ age + sex + health and lifestyle factors + GIANT PGS + MPS

Note: Models including health and lifestyle factors were only assessed in the LBC1936 due to the availability of phenotypic data. The health and lifestyle factors were determined by the PheWAS as described in the method below and are listed in Additional file 2: Table S7.

## Supplementary Figures


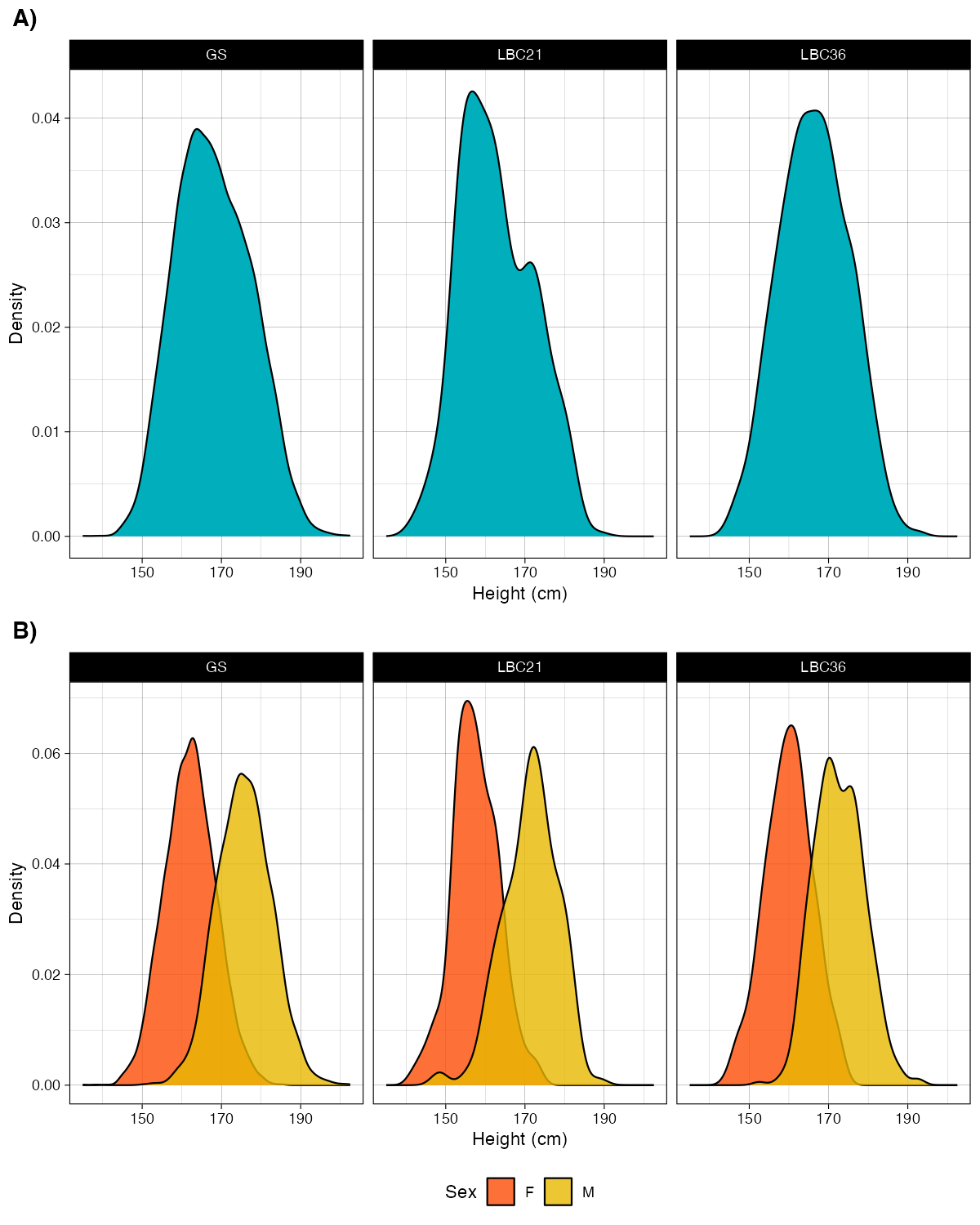


Fig. S1: Density plots of height in Generation Scotland (GS), the LBC1921 and LBC1936.

(A) Height and (B) height by sex for participants in GS (n=7,654), the LBCs (LBC21 n=435 and LBC36 n=861).


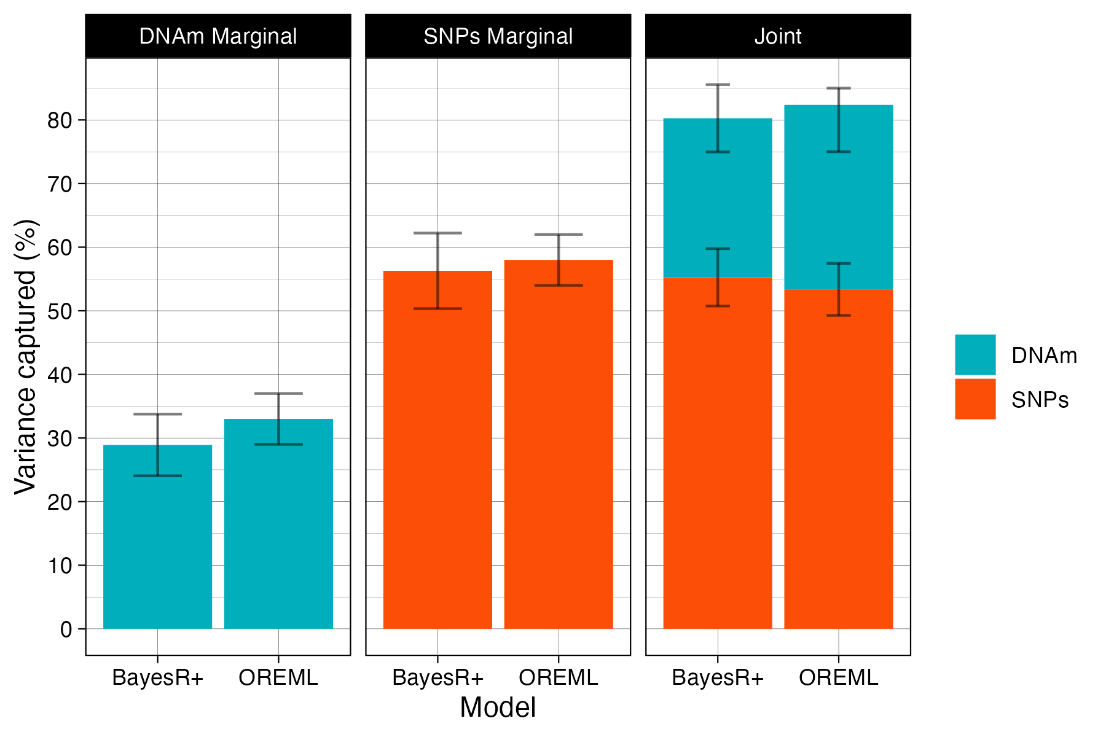


Fig. S2: The proportion of variance captured in height in Generation Scotland by genome-wide DNAm and SNPs by variance composition method.

Proportion of phenotypic variance in age-and-sex adjusted height captured by DNAm (blue) marginally, SNPs (red) marginally and DNAm and SNPs jointly. Variance estimates are presented for BayesR+ and OREML approaches. Error bars represent SE of the estimate.


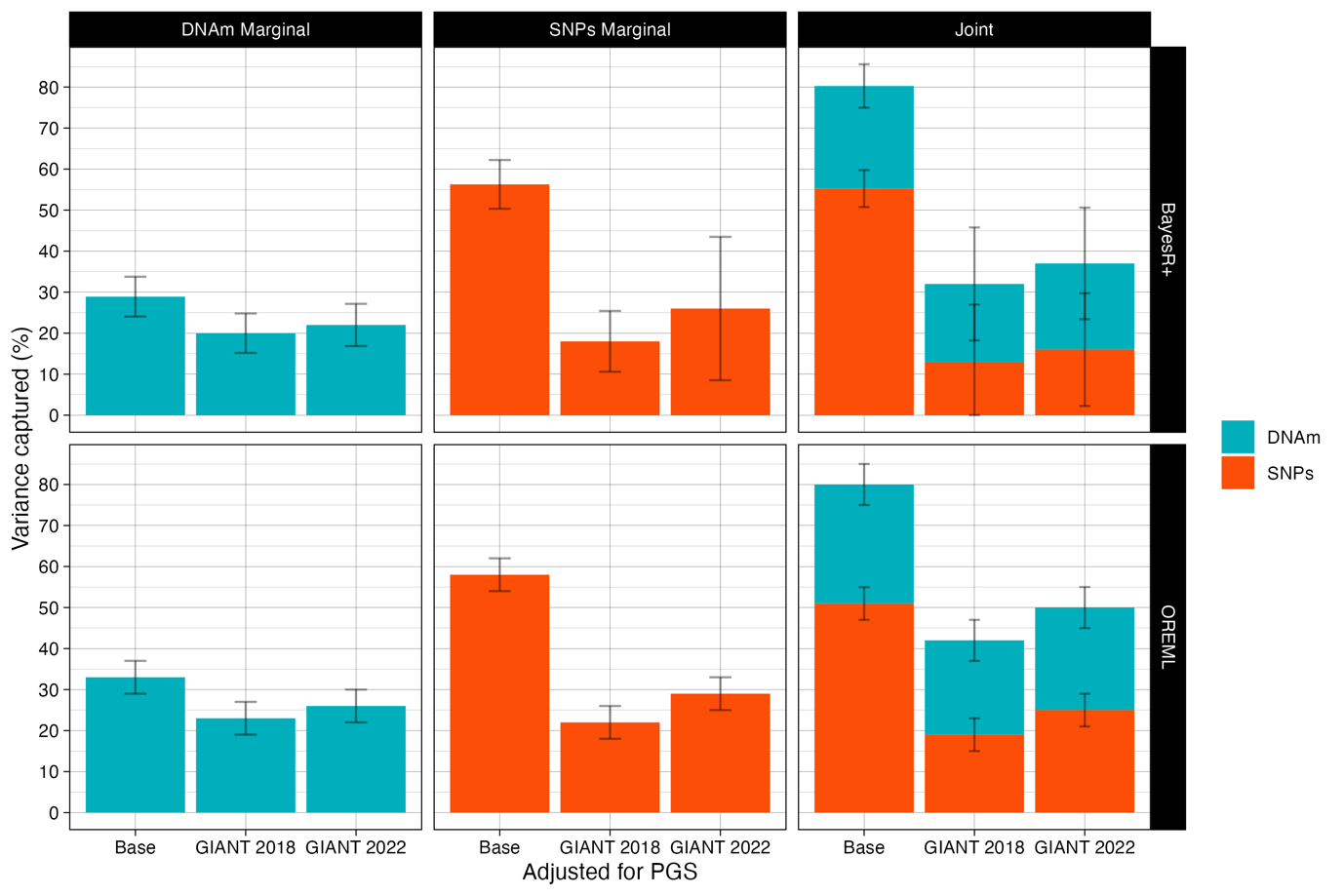


Fig. S3: Variance captured in height in Generation Scotland by DNAm and SNPs after adjusting for a PGS of height by method. Proportion of phenotypic variance in age-and-sex adjusted height captured by DNAm (blue) marginally, SNPs (red) marginally and DNAm and SNPs jointly. Variance estimates are presented for BayesR+ and OREML approaches. Models include base, addition of PGS from GIANT 2018 and GIANT 2022. Error bars represent SE of the estimate.


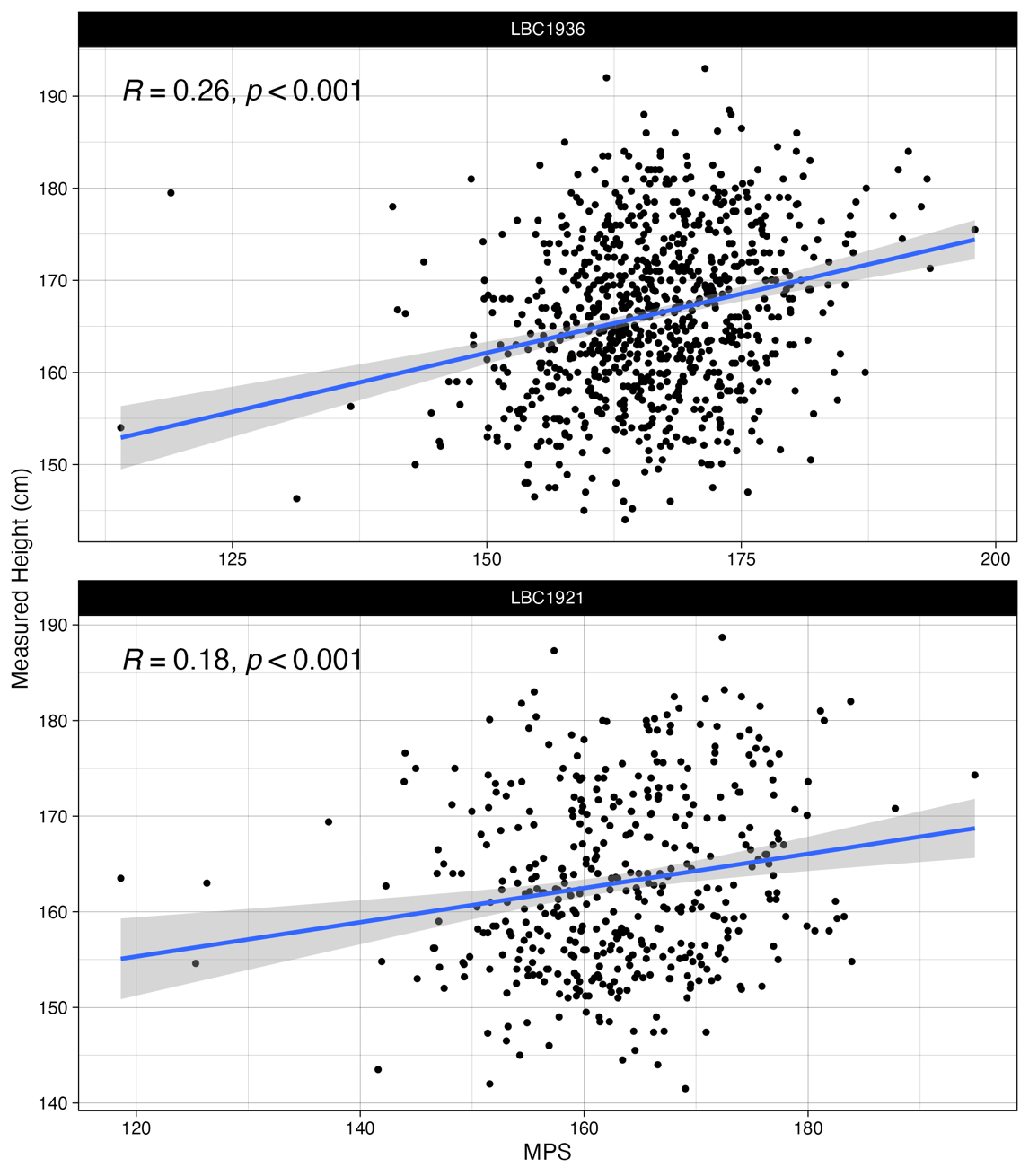


Fig. S4: Scatter Plot of height (measured) and MPS in the LBC1936 and LBC1921.

R indicates Pearson’s correlation and corresponding pvalue.

## References

1. Marioni RE, Campbell A, Scotland G, Hayward C, Porteous DJ, Deary IJ. Differential effects of the APOE e4 allele on different domains of cognitive ability across the life-course. European Journal of Human Genetics. 2016;24(6):919-23.

2. Yang J, Lee SH, Goddard ME, Visscher PM. GCTA: a tool for genome-wide complex trait analysis. American Journal Human Genetics. 2011;88(1):76-82.

3. The 1000 Genomes Project Consortium, Auton A, Abecasis GR, Altshuler DM, Durbin RM, Abecasis GR, et al. A global reference for human genetic variation. Nature. 2015;526(7571):68-74.

4. Amador C, Huffman J, Trochet H, Campbell A, Porteous D, Wilson JF, et al. Recent genomic heritage in Scotland. BMC Genomics. 2015;16(1):437.

5. Taliun D, Harris DN, Kessler MD, Carlson J, Szpiech ZA, Torres R, et al. Sequencing of 53,831 diverse genomes from the NHLBI TOPMed Program. Nature. 2021;590(7845):290-9.

6. Walker RM, McCartney DL, Carr K, Barber M, Shen X, Campbell A, et al. Data Resource Profile: Whole Blood DNA Methylation Resource in Generation Scotland (MeGS). medRxiv. 2024:2024.04.30.24306314.

7. Chen YA, Lemire M, Choufani S, Butcher DT, Grafodatskaya D, Zanke BW, et al. Discovery of cross-reactive probes and polymorphic CpGs in the Illumina Infinium HumanMethylation450 microarray. Epigenetics. 2013;8(2):203-9.

8. McCartney DL, Walker RM, Morris SW, McIntosh AM, Porteous DJ, Evans KL. Identification of polymorphic and off-target probe binding sites on the Illumina Infinium MethylationEPIC BeadChip. Genom Data. 2016;9:22-4.

9. Houseman EA, Accomando WP, Koestler DC, Christensen BC, Marsit CJ, Nelson HH, et al. DNA methylation arrays as surrogate measures of cell mixture distribution. BMC Bioinformatics. 2012;13(1):86.

10. Bollepalli S, Korhonen T, Kaprio J, Anders S, Ollikainen M. EpiSmokEr: a robust classifier to determine smoking status from DNA methylation data. Epigenomics. 2019;11(13):1469-86.

11. Deary IJ, Gow AJ, Pattie A, Starr JM. Cohort profile: the Lothian Birth Cohorts of 1921 and 1936. Int J Epidemiol. 2012;41(6):1576-84.

12. Deary IJ, Whiteman MC, Starr JM, Whalley LJ, Fox HC. The Impact of Childhood Intelligence on Later Life: Following Up the Scottish Mental Surveys of 1932 and 1947. Journal of Personality and Social Psychology. 2004;86(1):130-47.

13. Taylor AM, Pattie A, Deary IJ. Cohort Profile Update: The Lothian Birth Cohorts of 1921 and 1936. International Journal of Epidemiology. 2018;47(4):1042-r.

14. McRae AF, Powell JE, Henders AK, Bowdler L, Hemani G, Shah S, et al. Contribution of genetic variation to transgenerational inheritance of DNA methylation. Genome Biol. 2014;15(5):R73-R.

15. Shah S, McRae AF, Marioni RE, Harris SE, Gibson J, Henders AK, et al. Genetic and environmental exposures constrain epigenetic drift over the human life course. Genome research. 2014;24(11):1725-33.

16. Min JL, Hemani G, Davey Smith G, Relton C, Suderman M. Meffil: efficient normalization and analysis of very large DNA methylation datasets. Bioinformatics. 2018;34(23):3983-9.

17. Price ME, Cotton AM, Lam LL, Farré P, Emberly E, Brown CJ, et al. Additional annotation enhances potential for biologically-relevant analysis of the Illumina Infinium HumanMethylation450 BeadChip array. Epigenetics & chromatin. 2013;6(1):4-.

18. Anderson CA, Pettersson FH, Clarke GM, Cardon LR, Morris AP, Zondervan KT. Data quality control in genetic case-control association studies. Nature Protocols. 2010;5(9):1564-73.

19. Loh P-R, Danecek P, Palamara PF, Fuchsberger C, A Reshef Y, K Finucane H, et al. Reference-based phasing using the Haplotype Reference Consortium panel. Nature genetics. 2016;48(11):1443-8.

20. Boyd A, Golding J, Macleod J, Lawlor DA, Fraser A, Henderson J, et al. Cohort Profile: the 'children of the 90s'--the index offspring of the Avon Longitudinal Study of Parents and Children. Int J Epidemiol. 2013;42(1):111-27.

21. Fraser A, Macdonald-Wallis C, Tilling K, Boyd A, Golding J, Davey Smith G, et al. Cohort Profile: the Avon Longitudinal Study of Parents and Children: ALSPAC mothers cohort. Int J Epidemiol. 2013;42(1):97-110.

22. Northstone K, Ben Shlomo Y, Teyhan A, Hill A, Groom A, Mumme M, et al. The Avon Longitudinal Study of Parents and children ALSPAC G0 Partners: A cohort profile [version 2; peer review: 1 approved]. Wellcome Open Research. 2023;8(37).

23. Northstone K, Lewcock M, Groom A, Boyd A, Macleod J, Timpson N, et al. The Avon Longitudinal Study of Parents and Children (ALSPAC): an update on the enrolled sample of index children in 2019 [version 1; peer review: 2 approved]. Wellcome Open Research. 2019;4(51).

24. Harris PA, Taylor R, Thielke R, Payne J, Gonzalez N, Conde JG. Research electronic data capture (REDCap)--a metadata-driven methodology and workflow process for providing translational research informatics support. J Biomed Inform. 2009;42(2):377-81.

25. Relton CL, Gaunt T, McArdle W, Ho K, Duggirala A, Shihab H, et al. Data Resource Profile: Accessible Resource for Integrated Epigenomic Studies (ARIES). International Journal of Epidemiology. 2015;44(4):1181-90.

26. Delaneau O, Zagury JF, Marchini J. Improved whole-chromosome phasing for disease and population genetic studies. Nat Methods. 2013;10(1):5-6.

27. Das S, Forer L, Schönherr S, Sidore C, Locke AE, Kwong A, et al. Next-generation genotype imputation service and methods. Nature Genetics. 2016;48(10):1284-7.

28. McCarthy S, Das S, Kretzschmar W, Delaneau O, Wood AR, Teumer A, et al. A reference panel of 64,976 haplotypes for genotype imputation. Nat Genet. 2016;48(10):1279-83.

29. Trejo Banos D, McCartney DL, Patxot M, Anchieri L, Battram T, Christiansen C, et al. Bayesian reassessment of the epigenetic architecture of complex traits. Nature Communications. 2020;11(1):2865.

30. Zhang F, Chen W, Zhu Z, Zhang Q, Nabais MF, Qi T, et al. OSCA: a tool for omic-data-based complex trait analysis. Genome Biol. 2019;20(1):107.

31. Hatton AA, Hillary RF, Bernabeu E, McCartney DL, Marioni RE, McRae AF. Blood-based genome-wide DNA methylation correlations across body-fat- and adiposity-related biochemical traits. The American Journal of Human Genetics. 2023;110(9):1564-73.

32. Lee SH, Wray NR, Goddard ME, Visscher PM. Estimating missing heritability for disease from genome-wide association studies. American Journal of Human Genetics. 2011;88(3):294-305.

33. Lloyd-Jones LR, Zeng J, Sidorenko J, Yengo L, Moser G, Kemper KE, et al. Improved polygenic prediction by Bayesian multiple regression on summary statistics. Nature Communications. 2019;10(1):5086.

34. Yengo L, Vedantam S, Marouli E, Sidorenko J, Bartell E, Sakaue S, et al. A saturated map of common genetic variants associated with human height. Nature. 2022;610(7933):704-12.

35. Chang CC, Chow CC, Tellier LC, Vattikuti S, Purcell SM, Lee JJ. Second-generation PLINK: rising to the challenge of larger and richer datasets. Gigascience. 2015;4:7.

36. Yengo L, Sidorenko J, Kemper KE, Zheng Z, Wood AR, Weedon MN, et al. Meta-analysis of genome-wide association studies for height and body mass index in ∼700000 individuals of European ancestry. Human Molecular Genetics. 2018;27(20):3641-9.

37. Zeng J, de Vlaming R, Wu Y, Robinson MR, Lloyd-Jones LR, Yengo L, et al. Signatures of negative selection in the genetic architecture of human complex traits. Nature Genetics. 2018;50(5):746-53.

38. Hillary RF, McCartney DL, Smith HM, Bernabeu E, Gadd DA, Chybowska AD, et al. Blood-based epigenome-wide analyses of 19 common disease states: A longitudinal, population-based linked cohort study of 18,413 Scottish individuals. PLoS Med. 2023;20(7):e1004247.

39. Song N, Hsu CW, Pan H, Zheng Y, Hou L, Sim JA, et al. Persistent variations of blood DNA methylation associated with treatment exposures and risk for cardiometabolic outcomes in long-term survivors of childhood cancer in the St. Jude Lifetime Cohort. Genome Med. 2021;13(1):53.

40. Hillary RF, Ng HK, McCartney DL, Elliott HR, Walker RM, Campbell A, et al. Blood-based epigenome-wide analyses of chronic low-grade inflammation across diverse population cohorts. Cell Genomics. 2024;4(5):100544.

41. Christiansen C, Castillo-Fernandez JE, Domingo-Relloso A, Zhao W, El-Sayed Moustafa JS, Tsai PC, et al. Novel DNA methylation signatures of tobacco smoking with trans-ethnic effects. Clin Epigenetics. 2021;13(1):36.

42. Gadd DA, Hillary RF, McCartney DL, Shi L, Stolicyn A, Robertson NA, et al. Integrated methylome and phenome study of the circulating proteome reveals markers pertinent to brain health. Nat Commun. 2022;13(1):4670.
